# The Scorpion Toxin Tf2 from *Tityus fasciolatus* Promotes Na_v_1.3 Opening

**DOI:** 10.1371/journal.pone.0128578

**Published:** 2015-06-17

**Authors:** Thalita S. Camargos, Frank Bosmans, Solange C. Rego, Caroline B. F. Mourão, Elisabeth F. Schwartz

**Affiliations:** 1 Departamento de Ciências Fisiológicas, Laboratório de Toxinologia, Universidade de Brasília, Brasília, DF, Brazil; 2 Department of Physiology, Johns Hopkins University—School of Medicine, Baltimore, MD, United States of America; 3 Solomon H. Snyder Department of Neuroscience, Johns Hopkins University—School of Medicine, Baltimore, MD, United States of America; The Hebrew University Medical School, ISRAEL

## Abstract

We identified Tf2, the first β-scorpion toxin from the venom of the Brazilian scorpion *Tityus fasciolatus*. Tf2 is identical to Tb2-II found in *Tityus bahiensis*. We found that Tf2 selectively activates human (h)Na_v_1.3, a neuronal voltage-gated sodium (Na_v_) subtype implicated in epilepsy and nociception. Tf2 shifts hNa_v_1.3 activation voltage to more negative values, thereby opening the channel at resting membrane potentials. Seven other tested mammalian Na_v_ channels (Na_v_1.1-1.2; Na_v_1.4-1.8) expressed in *Xenopus* oocytes are insensitive upon application of 1 μM Tf2. Therefore, the identification of Tf2 represents a unique addition to the repertoire of animal toxins that can be used to investigate Na_v_ channel function.

## Introduction

The *Tityus fasciolatus* scorpion is found in the Cerrado biome in Brazil and is phylogenetically related to *Tityus serrulatus*, a scorpion responsible for many envenomation cases [1,2]. In contrast to *T*. *serrulatus* however, getting stung by *T*. *fasciolatus* is less likely since this scorpion is predominantly found in termite hills and rarely moves into human habitats. The venom toxicity of *T*. *fasciolatus* is considered moderate with a median lethal dose (LD_50_) of 56.65μg/mouse [3]. So far, Tf4 is the only peptide purified from this venom and based on its amino acid sequence and activity in sucrose gap assays in frog nerves, it is classified as an α-scorpion toxin acting on voltage-gated sodium (Na_v_) channels [4]. Since their pivotal role in cellular excitability makes them an ideal target to incapacitate prey or predators, scorpion venoms have evolved to contain a plethora of toxins targeting Na_v_ channels [5].

α-Scorpion toxins primarily interfere with the voltage-drive activation process of the domain IV voltage sensor within the channel [6–8]. Since the primary role of this region is to inactivate the channel a few milliseconds after it opens (*i*.*e*. fast inactivation) [9–17], α-scorpion toxins slow down fast inactivation resulting in a persistent inward Na^+^ current when the channel should no longer conduct. In contrast to α-scorpion toxins, β-scorpion toxins activate Na_v_ channels at voltages where they should normally be closed without affecting fast inactivation [18,19]. In general, the working mechanism underlying β-scorpion toxin activity involves stabilizing the voltage sensor in domains I, II, or III in an activated state to facilitate Na_v_ channel activation [9,18–23]. Given their potency and exquisite selectivity, both toxin groups have been used extensively to investigate Na_v_ channel function [5]. However, very few toxins have been identified that target just one of the nine Na_v_ channel isoforms found in mammals. Such a tool would be beneficial to probe the physiological role of a particular isoform in an organism [24].

While testing the venom of *T*. *fasciolatus* on an array of Na_v_ channel isoforms, we came across a fraction that selectively activated human (h)Na_v_1.3, a neuronal subtype believed to be involved in epilepsy as well as pain perception after channel upregulation due to spinal cord injury [25–27]. Further fraction purification resulted in the identification of Tf2, the first β-scorpion toxin isolated from the venom of *T*. *fasciolatus*. Tf2 can shift hNa_v_1.3 activation voltage to much more negative values, effectively opening the channel at resting membrane potentials. Remarkably, seven other tested mammalian Na_v_ channels (Na_v_1.1–1.2; Na_v_1.4–1.8) are insensitive to Tf2 upon application of an identical quantity. As such, the identification of Tf2 represents a distinct addition to the range of animal toxins that can be used to investigate hNa_v_1.3 function.

## Materials and Methods

### Animal and venom collection

Specimens of *T*. *fasciolatus* were collected in Brasilia, Federal District, Brazil (15°46’S 47°50’W), under license n° 19138–1 (IBAMA- Instituto Brasileiro do Meio Ambiente e dos Recursos Naturais). They were kept at appropriate facilities at the University of Brasilia with food and water *ad libitum*. Scorpions were submitted to electrical stimulation close to the telson, respecting a 30 day interval. The venom was collected, solubilized in a watery trifluoroacetic acid (TFA) 0.12% solution, and centrifuged at 15,000 × g for 15 min. The supernatant was collected and quantified at 280 nm and dried. Hereafter, the material was kept at -20°C.

### Toxin purification

Dried *T*. *fasciolatus* venom was dissolved in purified water and separated by RP-HPLC (reverse-phase high performance liquid chromatography) using a C18 analytical column (Synergi Fusion RP 4μ 80 Å 250 × 4.6 mm Phenomenex, Inc., USA), using a linear gradient from 0% solvent A (0.12% TFA in water) to 60% solvent B (0.10% TFA in acetonitrile) over 60 min at a flow rate of 1 mL/min, with detection at a wavelength of 216 and 280nm. The optimized chromatography protocols used to purify Tf2 consisted of three extra steps with a gradient from 25% to 45% solvent B in 40 min with the two last steps performed at 45°C. The purity of the toxin was verified by MALDI-TOF mass spectrometry and by the area under curve on HPLC and was higher than 97%.

### Molecular mass and amino acid sequence determination

The molecular mass and purity of the sample was checked on an Ultraflex III MALDI TOF mass spectrometer (Bruker Daltonics, Germany). The sample was dissolved in an α-cyano-4-hydroxycinnamic acid matrix (1:3, v:v), spotted onto the MALDI target plate and dried at room temperature for 15 min and analyzed in positive reflected and linear modes. External calibration was preceded by a Peptide Calibration Standard for mass spectrometry (Bruker Daltonics) for reflector mode and Protein 1 mixture (Bruker Daltonics) for linear mode. Spectra were analyzed with FlexAnalysis 3.3 (Bruker Daltonics, Germany). In order to check the accurate monoisotopic molecular mass of Tf2, a micrOTOF-QII (Bruker Daltonics) equipped with an orthogonal electrospray ionization source (ESI) operated in the positive mode was used. Sample was diluted in variable concentrations of 1% formic acid in a water/acetonitrile mixture (1:1, v:v) and applied to the mass spectrometer source by direct infusion.

The complete amino acid sequence was obtained from a transcriptomic library performed with RNA isolated from the *T*. *fasciolatus* venom gland (Hiseq, Illumina, USA). The signal peptide was identified using SignalP 4.1 server [28], and the amidation signal site was determined using insights gained from previous characterized toxins. To verify the peptide sequence, the first 15 amino acid residues were sequenced by automated Edman degradation on a Beckman (Palo Alto, CA) LF 3000 protein peptide sequencer. Similarity searches were performed using blastp (http://www.ncbi.nlm.nih.gov/blast) with an *e* value cutoff set to <10^–5^ to identify putative functions. ClustalW version 2.0 [29] was used for sequence alignments and calculation of the percentage of identity between paired sequences. The consensus sequence of alignment was colored using Chroma software [30]. The protein reported in this work is deposited in UniProt Knowledgebase as entry C0HJM9 and nucleotide sequence is deposited in EMBL-EBI as entry LN606597.

### Molecular modeling

Model construction of Tf2, Ts2 and CssIV was performed by the fold recognition approach for identifying potential templates using the Phyre2 server [31]. This server is able to identify homologs using distant sequences obtained from algorithms such as PSI-BLAST and HMM [32,33]. The template with the highest score, Ts1 (Protein Data Bank ID 1NPI, 1.16 Å resolution), was chosen for both Tf2 and Ts2 model construction, with 100% coverage and 75% / 74% identity, respectively [34]; and Cn2 template (PDB ID 1CN2, 2.21 Å resolution) [35] was chosen for CssIV modeling, with 100% coverage and 83% identity. Additionally, CssII and AaHII three-dimensional structures (PDB ID 2LI7 [36] and 1PTX [37], respectively), were used in the structural and electrostatic potential surface comparison with Tf2. The alpha toxin AaHII was used in order to emphasize the structural differences between alpha and beta sodium scorpion toxins. Structural alignment of all proteins (Tf2, Ts2, Ts1, CssIV, CssII, and AaHII) was performed using the plugin MultiSeq by Visual Molecular Dynamics (VMD) [38] and the electrostatic potential surfaces were calculated using CCP4 Molecular Graphics Program [39] by the built-in Poisson-Boltzmann method.

### Electrophysiology using *Xenopus* oocytes

Human (h)Na_v_1.4, hNa_v_1.5, and rat (r)Na_v_1.8 were a gift from Peter Ruben (Simon Fraser University, Canada), Chris Ahern (University of Iowa, USA), and John Wood (UCL, UK), respectively. hNa_v_1.1–1.3, hNa_v_1.6–1.7 were obtained from Origene Technologies, Inc. (MD, USA). Accession numbers are NM_001165963.1 (hNa_v_1.1), NM_021007.2 (hNa_v_1.2), NM_006922.3 (hNa_v_1.3), NM_000334 (hNa_v_1.4), NM_198056 (hNa_v_1.5), NM_014191.2 (hNa_v_1.6), NM_002977.2 (hNa_v_1.7), and NM_017247.1 (rNa_v_1.8). cRNA of hNa_v_1.1–1.7 and rNa_v_1.8 was synthesized using T7 polymerase (mMessage mMachine kit, Life technologies, USA) after linearizing the fully-sequenced DNA with appropriate restriction enzymes. Channels were expressed together with the hβ1 subunit (Origene Technologies, Inc., NM_001037.4) (1:5 molar ratio) in *Xenopus laevis* oocytes (acquired from Xenopus one, USA) that were incubated at 17°C in 96 mM NaCl, 2 mM KCl, 5 mM Hepes, 1 mM MgCl_2_, 1.8mMCaCl_2_, and 50 g/mL gentamycin (pH 7.6 with NaOH) for 1–4 days after cRNA injection, and then were studied using two-electrode voltage-clamp recording techniques (OC-725C; Warner Instruments) with a 150-μl recording chamber. Data were filtered at 4 kHz and digitized at 20 kHz using pClamp10 software (Molecular Devices, USA). Microelectrode resistances were 0.5–1 MΩ when filled with 3 M KCl. The external recording solution contained 100 mM NaCl, 5 mM Hepes, 1 mM MgCl_2_, and 1.8 mM CaCl_2_ (pH 7.6 with NaOH). All experiments were performed at room temperature (22°C). Leak and background conductance, identified by blocking the channel with tetrodotoxin (Alomone Laboratories, Israel), were subtracted for all Na_v_ channel currents. The G-V curves were plotted with OriginPro8 (3<n<6), normalized, and fitted with the Boltzmann equation. A t-test was carried out to calculate the statistical relevance of each experiment with p<0.05.

## Results

### Toxin purification and sequence determination

**Fig 1:**
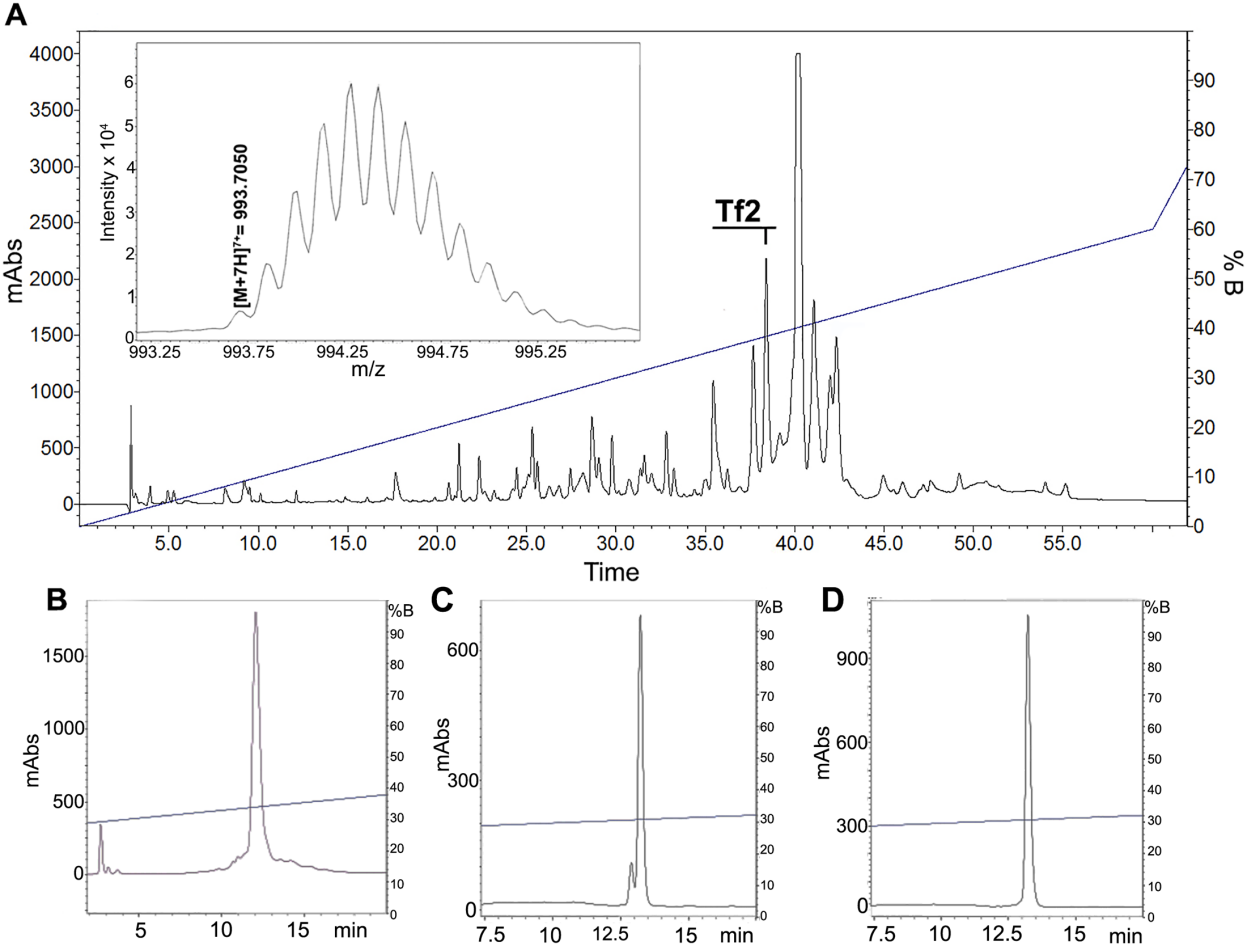
Purification and molecular mass determination of Tf2. (A) Chromatography by RP-HPLC of 1 mg of *T*. *fasciolatus* crude venom. Fractionation was performed on a C18 analytical column, using a linear gradient from 0% solvent A (0.12% TFA in water) to 60% solvent B (0.10% TFA in acetonitrile) over 60 min at a flow rate of 1 mL/min, with detection at a wavelength of 216 and 280nm. The component eluted at 38.5% of acetonitrile corresponds to Tf2. (B-D) Three additional chromatographic protocols performed to obtain pure Tf2. (B) Linear gradient of B solvent, from 25 to 45% B in 40 minutes, at room temperature (22°C). (C) Linear gradient of B solvent, from 25 to 45% of B in 40 minutes, at 45°C. (D) Linear gradient of B solvent, from 30 to 40% of B in 40 minutes, at 45°C. Inset on (A) shows mass spectrometry analysis of Tf2 by micrOTOF-Q II, presenting the monoisotopic distribution of the +7 charged ion ([M+7H]^7+^ = 993.7050), which is equivalent to [M+H]^+^ 6949.9350 Da.

**Fig 2:**
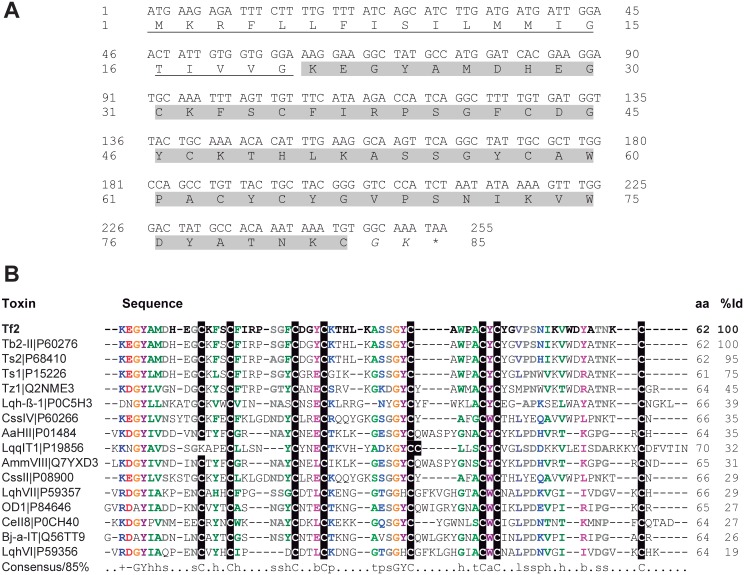
Tf2 sequence and alignment with scorpion Na_v_ channel toxins. (A) The nucleotide sequence of Tf2 was obtained by HiSeq (Illumina, USA). Signal peptide is underlined, mature peptide is highlighted in gray, and the amidation set point is marked in italic. (B) Multiple sequence alignment of Tf2 with other Na_v_ channel toxins. Toxins are presented by their short names and UniProt KB codes. Capital letters denote amino acids. Lower-case letters denote: h, hydrophobic; s, small; b, big; p, polar; t, tiny; a, aromatic; l, aliphatic. Positive (+) and negative (-) amino acid residues that are part of the consensus sequence are also colored. Cys residues are shaded in black. aa means amino acid residues, and %Id is the percentage of sequence identity with Tf2.

1 mg of *T*. *fasciolatus* venom was divided into 60 fractions using reversed-phase HPLC with a linear acetonitrile gradient and Tf2 was collected at 38.5% of acetonitrile (Fig 1A). Next, three additional purification steps with varying degrees of acetonitrile gradients were performed to obtain the final pure toxin (Fig 1B–1D). The amino acid sequence was deduced by a combination of Edman sequencing and a RNA library (unpublished data) obtained from RNA extracted from the venom gland. The nucleotide sequence that codes for Tf2 contains 255 nucleotides, including the stop codon, and the translated peptide contains a signal peptide with 20 amino acid residues, and a 62 residue mature peptide (Fig 2A). Na_v_ channel scorpion toxins containing a GK at the C-terminal end are frequently amidated [40], an important feature for toxins such as Ts1 and CssII since this post translational modification increases their biological activity [41,42]. Taking protein amidation into account (-0.98 Da), as well as the presence of four disulfide bridges, the theoretical monoisotopic molecular mass is [M+H]^+^ 6,950.0302 Da, which corresponds with the experimental monoisotopic molecular mass of [M+H]^+^ 6,949.9350 Da (or [M+7H]^7+^ = 993.7050) obtained by using the micrOTOF QII (see inset Fig 1A). The calculated error between theoretical and experimental molecular mass, using the fifth isotope of the isotopic series, was 0.6 ppm.

Aligning Tf2 with other toxins found in databases (Fig 2B) reveals 100% identity with Tb2-II, a toxin from the South American scorpion *Tityus bahiensis* that is lethal upon injection into mammals and insects [43]. However, the molecular target of Tb2-II has not been identified yet. Tf2 is also 95% identical (3 amino acid difference) to Ts2, a toxin from *T*. *serrulatus* venom which inhibits fast inactivation of Na_v_1.2, Na_v_1.3, Na_v_1.5, Na_v_1.6, and Na_v_1.7, but does not affect Na_v_1.4 or Na_V_1.8 [44,45]. Interestingly, Ts2 also shifts the voltage-dependence of activation of Na_v_1.3 to more negative potentials suggesting that multiple voltage sensors may be targeted [9]. Sequence alignments were conducted with toxins that were tested on heterologously expressed-voltage gated ion channels as well as representative examples of toxins of which 3D structures have been determined. Such an alignment could provide insights into the features that are important for the biological activity of Tf2. For example, in Tf2, Tb2-II, and Ts2, a conserved cluster of aromatic residues formed by Y^4^, Y^37^, Y^44^, Y^46^, W^40^ and W^55^ was observed (Fig 2B). This is known to be as an important feature for the activity of β-scorpion toxins [46,47].

### Homology modeling

For structural analysis, the NMR structures of Ts1, CssII, and AaHII, were compared with the homology models of Tf2, Ts2, and CssIV. Tf2 and Ts2 models were constructed using Ts1 as template whereas the CssIV model was obtained using Cn2 as template. Since the effect of different amino acid mutations is well studied in the β-scorpion toxins CssII and CssIV, we chose these as reference molecules to better understand the structure-function relationship of Ts1, Ts2 and Tf2 [48,49]. The α-toxin AaHII was chosen as a representative example of α-scorpion toxins to evaluate the differences with this particular family [42,50].

**Fig 3:**
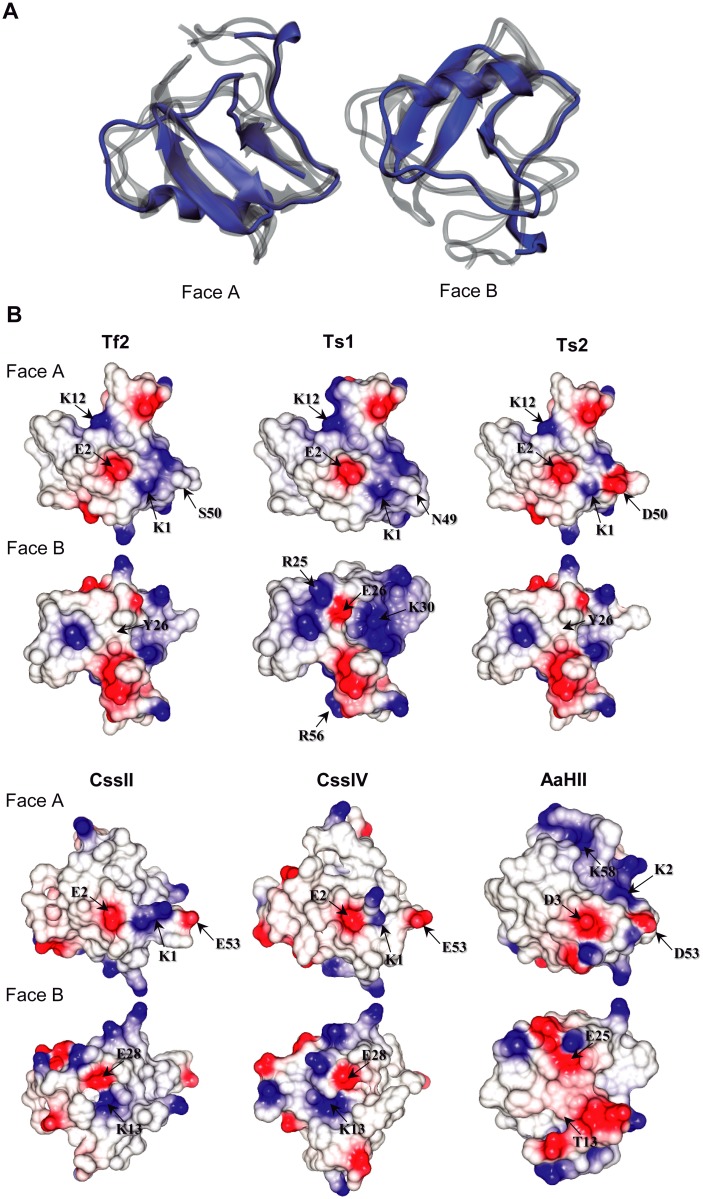
Structural comparison between Tf2 and other Na_v_ channel toxins. (A) Structural alignment between Tf2 (in blue) and five other Na_v_ channel scorpion toxins—Ts1, Ts2, CssII, CssIV, and AaHII (in gray). (B) Comparison of electrostatic potentials between the toxins Tf2, Ts1, Ts2, CssII, CssIV, and AaHII. The figure shows charge distribution along the toxin surface, divided into faces A and B. Shown in red are acidic residues whereas blue represents basic residues; in white, neutral regions are shown.

Resulting structural alignments reveal a superposition of secondary structures, an observation that is typical for scorpion toxins targeting Na_v_ channels (Fig 3A) [46]. However, the electrostatic potential diverges when comparing the structures of toxins Ts1, Ts2 and Tf2 with the structures of CssII, CssIV and AaHII (Fig 3A and 3B). This feature has been suggested before to be responsible for the difference in target sensitivity [51–55]. All toxins possess a centrally-located residue with negative electrostatic potential (Fig 3B, face A), corresponding to a glutamic acid (E^2^, present in all β-scorpion toxins, including Ts2) or an aspartic acid (D^3^, present in the α-scorpion toxin AaHII). Another feature observed in all analyzed structures is the presence of a positive region close to the negative core, *i*.*e*. a lysine residue situated at position 1 for the β-scorpion toxins, and position 2 for the α-scorpion toxin AaHII. Comparing Tf2 to other scorpion toxins suggests that positively charged groups at positions 1 and 12 (K^12^ to Tf2, Ts1 and Ts2, K^13^ to CssII and CssIV), as well a negative charge at position 2 are likely determinants of β-scorpion toxin specificity [47]. In α-scorpion toxins, the positively charged residue is commonly found in position 58 instead of 12 (K^58^ in AaH2, Fig 3) [47], whereas with Ts2 has a K^12^ and A^58^ (Fig 2B) [44]. However, all β-scorpion toxins analyzed here possess these characteristics, yet Tf2 acts primarily on hNa_v_1.3 whereas the others do not.

The primary sequences of Tf2 and Ts2 differ in three residues (S^20^A, S^50^D and N^51^H) (Fig 2B), among which only the substitution at position 50 induces a significant change in the electrostatic potential of the molecule. The presence of this aspartic acid generates a negative potential in Ts2, which is also absent in Ts1 (N^49^ residue in the equivalent position) (Fig 3B, face A). When comparing the structures of Ts1 to those from Tf2 and Ts2, we observe an increase of positively charged regions, originating from an arginine at positions 25/56 and a lysine at position 30 (Fig 2B, face B). The high degree of sequence and structural conservation of charged groups observed in Tf2, Ts1 and Ts2 at face A suggests that this structural pattern may have an important role in the recognition and specificity towards particular Na_v_ channel isoforms.

CssII, CssIV, and AaHII also have an acid residue in a similar acidic region of D^50^ found in Ts2 (E^53^, E^53^ and D^53^, respectively) (Figs 2B and 3B). Although presenting a different electrostatic potential in comparison to Ts2 and Tf2, all these toxins have small amino acid residues in equivalent positions, as observed in the consensus alignment (Fig 2B) and at face A of structures (Fig 3B). As expected by their high degree of similarity, face B of Tf2 and Ts2 have similar electrostatic potentials. In comparison, face B of Ts1 is more positively charged due to the presence of residues R^25^, K^30^ and R^56^. Additionally, an acidic region, usually at position E^28^, can be visualized at face B in almost all these proteins, which is known as a key pharmacophore region in scorpion toxins targeting Na_v_ channels [46]. However, both Tf2 and Ts2 present an aromatic residue at this position (Y^26^; see Fig 3B, face B).

### Tf2 activity on mammalian Na_v_ channels expressed in oocytes

**Fig 4:**
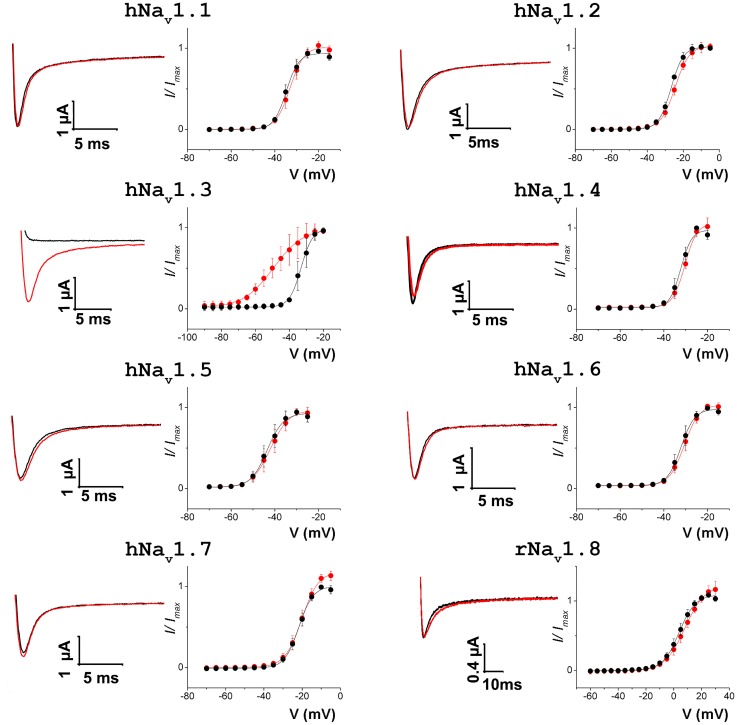
Effect of Tf2 on Na_v_ channel isoforms expressed in *X*. *laevis* oocytes. Shown on the left in each column is a representative trace of experimental Na^+^ currents obtained by depolarizing the membrane to a suitable voltage from a holding potential of -90mV, at -25mV for Na_v_ 1.1–1.2, 1.4–1.7, at -40mV for Na_v_1.3, and at 20mV for Na_v_1.8. Shown on the right in each column is a deduced conductance (G)—voltage (V) relationship before (black) and after (red) the application of 1μM Tf2. This concentration only influences the activation of hNa_v_1.3. Data is shown as mean ± SEM of n > 3.

To examine the biological activity of Tf2, we used the two-electrode voltage-clamp technique to measure sodium currents from 8 mammalian Na_v_ channel isoforms expressed in *X*. *laevis* oocytes before and after toxin addition. At 1 μM, Tf2 does not influence currents generated by hNa_v_1.1–1.2, 1.4–1.7, and rNa_v_1.8 (Fig 4); however, hNa_v_1.3 activation is drastically influenced. At 1μM, Tf2 shifts the voltage-dependence of activation by ~16 mV (V_1/2_ from -33.1 ± 0.2mV to -49.3 ± 0.5mV; slopes 3.4 ± 0.2 and 8.9 ± 0.5). To a certain extent, this effect resembles that of Ts2, which promotes opening of rNa_v_1.3 when applying 1 μM toxin [44]. However, Ts2 also hampers fast inactivation of this and other rNa_v_ channel isoforms whereas Tf2 does not appear to influence activation or fast inactivation of other Na_v_ channel isoforms. Thus, Tf2 activity seems to be unique among β-scorpion toxins and may be valuable to further hNa_v_1.3 research.

## Discussion

In this work, we report the discovery and characterization of Tf2, the first β-scorpion toxin from the venom of *T*. *fasciolatus*. In contrast to related β-scorpion toxins [34,36,44,45,49], Tf2 preferentially potentiates hNa_v_1.3 opening, resulting in a dramatic shift in activation voltage. As a result, delivery of 1 μM Tf2 to Na_v_1.3 expressed in neurons may cause the channel to open at resting membrane voltages, an effect that would be detrimental to the envenomed organism.

Given its exquisite selectivity pattern, it is interesting to consider the mechanism underlying Tf2 activity. When comparing the amino acid sequence of Tf2 and Ts2, only three substitutions can be found (S^20^A, S^50^D, and N^51^H). As a result of the substitution S^50^D, Ts2 has a lower overall charge that may affect toxin ability to interact with its binding site, which may be located within the lipid membrane. In Ts1, the residue W^50^, equivalent to the residues H^51^ in Ts2 or N^51^ in Tf2, is crucial for toxin activity [47]. Thus, it is possible that the different effects between Tf2 and Ts2 on Na_v_ channels are due to this substitution or due to the charge difference caused by S^50^D. Hence, in order to elucidate the different effects of Tf2 and Ts2, it will be interesting to perform site-directed mutagenesis and test both Tf2 and Ts2 under similar electrophysiological conditions.

Overall, Tf2 possess all the important characteristics mentioned by Polikarpov *et al*., [47] that are relevant for the activity of Na_v_ channel toxins, such as a positive electrostatic potential at position 1 (K^1^), a negative potential at position 2 (E^2^), a positively charged group at position 12 (K^12^) and a solvent-exposed aromatic core (Y^4^, Y^37^, Y^44^, Y^46^, W^40^ and W^55^). In all presented structures, the central negative residue (E^2^ or D^3^) is surrounded by the aromatic core. At face B, Tf2 and Ts2 possess an Y^28^ instead of the E^28^. As discussed by Cohen et al., [49], the charge neutralization of this acidic residue resulted in a three order decrease of CssIV binding affinity. Maybe this neutralization could represent a clue for the high specificity of Tf2 to hNav1.3. CssII and CssIV also possess these characteristics, but the basic residue at position 12 is actually at position 13, which is located at face B. Despite of the different face, these peptides maintain the β-scorpion toxin activity. Instead of a K^12^, the toxin AaHII has a lysine residue at position 58, as a proposed conserved feature in α-scorpion toxins.

**Fig 5:**
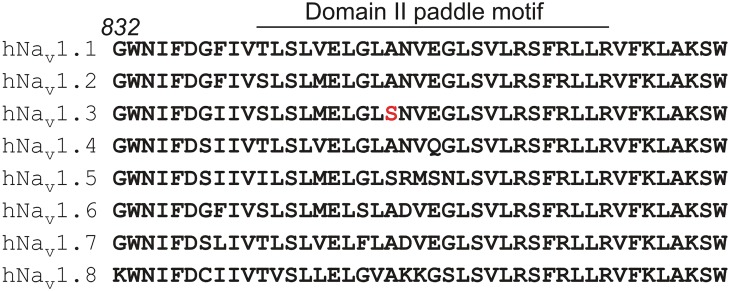
Sequence alignment of the domain II paddle motif in 8 mammalian Na_v_ channel isoforms. Figure shows a sequence alignment of the domain II paddle motif as found in 8 mammalian Na_v_ channel isoforms. As a reference, the number in italic indicates the coordinates of the first Gly residue in hNa_v_1.1. Although the Ile in hNa_v_1.3 (position 830 according to hNa_v_1.3 coordinates, 840 according to hNa_v_1.1 coordinates) differs from the Phe found in other neuronal isoforms, this residue is not present within the paddle motif and may not be accessible to Tf2. The Ser at position 842 (hNa_v_1.3 coordinates—indicated in red) is unique among hNa_v_1.1–1.3.

In general, β-scorpion toxins interact primarily with the S3b-S4 paddle motif within the domain II voltage sensor in Na_v_ channels [9,18–23]. However, a sequence alignment of this region reveals a high degree of amino acid conservation between hNa_v_1.1-r1.8 (Fig 5). Even though the Ser at position 842 in hNa_v_1.3 is unique among most Na_v_ channel isoforms and may therefore contribute to subtype selectivity of the toxin, a Ser is also found in hNa_v_1.5 which is insensitive to Tf2. However, hNa_v_1.5 differs in the four ensuing residues (Arg/Met/Ser/Asn) from hNa_v_1.3. In contrast to α-scorpion toxins for which an interaction with the paddle motif is the domain IV voltage sensor is sufficient to exert their effect [9,56–59], it is important to mention that residues outside of the domain II paddle motif may be required for activity of β-scorpion toxins [8,18,19,21,51,60–62]. As such, the Na_v_ channel isoform selectivity of Tf2 may be determined by amino acids in regions outside of the anchoring point in the domain II paddle motif. Further mutagenesis studies will therefore help delineate the complete binding site of Tf2. Finally, it is also worth considering that the extent of the effect on hNa_v_1.3 as observed in the *X*. *laevis* system, may differ in mammalian neurons due to a different membrane lipid composition or the presence of β-subunits [63,64]. Altogether, the distinct selectivity pattern of Tf2 may be a powerful tool to investigate the role of hNa_v_1.3 in epilepsy or nociception after spinal cord injury [25–27]. Moreover, the dramatic shift in channel activation voltage in the presence of Tf2 could be exploited in drug screening assays (*e*.*g*. FLIPR) in which voltage-control is impractical to open the channel in the presence of therapeutics [24,65].
